# Annotations

**Published:** 1911-07-29

**Authors:** 


### ANNOTATIONS.

### Suicides in the Hot Weather.

The reference to cases of suicide said to be consequent upon the heat draws attention to the fact that people of nervous temperament may be seriously affected by various climatic conditions. Certain seasons of the year also appear to influence similar temperaments. The neurasthenic is generally most incapable of mental or physical work in the spring. That there are forces at work at some seasons of the year which may manifest themselves by producing some effects upon the body is evident from the presence, virtually only in spring or autumn, of such a disease as erythema nodosum. Possibly the fact that spasmus nutans almost invariably commences in the early months of the year may also be due to some curious force acting on the nervous system, though a much more simple explanation for the origin of the disease at this time of year has been suggested. People susceptible to some irregularity of the heart seem to be frequently most affected in cases of unsettled weather, especially, to use their words, when "there is thunder in the air." Apparently, also, some dyspeptics suffer chiefly in similar weather. This upsetting of the digestion we must suppose to be due to some effect of the weather upon the vasomotor system of the abdomen. It is curious, too, how different may be the feelings of various people in the same wind. One person will feel bright and energetic when the wind is in the east. Another will say that it "takes all the life out of him."

### "The Repentance of Mr. Burns."

Under this heading a newspaper article attributes to Mr. Burns a sudden change of front with regard to the importance of milk in the spread of tuberculosis. It is no part of our duty to defend a Cabinet Minister, but the remarks we have seen quoted of Mr. Burns seem to show that he has adopted a common-sense attitude with regard to the causes of tuberculosis. It is necessary that we should have pure milk, but if all tubercle bacilli are for ever abolished from milk the diminution in the death-rate from tuberculosis will be very small. Tuberculosis due to the bovine bacillus is virtually limited to infants and young children, and only a small percentage of tuberculosis in these younger members of the community is due to the bovine bacillus. It is even possible that the Royal Commission may have over-estimated the number of cases infected with tuberculosis by means of milk. We have heard on good authority from those engaged in medical work in China that all the forms of tuberculosis from which our English children suffer are common in children in China living in districts where they receive no milk whatever. Obviously, therefore, in China milk is not responsible for tuberculosis in children, and the abolition of milk from the diet or tubercle bacilli from the milk given to children in England unfortunately would not save them from tuberculosis. Any known avenue by which an enemy may approach must be guarded, but it is foolish to imply that the closing of one avenue should give a great sense of security. There is much to learn about the life history of the tubercle bacillus, and further knowledge may aid to prevent its attacks upon us, but meanwhile it is reasonable to do all we can to bring to our aid the enemies of the bacillus, sunlight and air.

### The Small-pox Outbreak.

The Metropolitan Asylums Board has issued a report concerning the recent outbreak of small-pox in London. The statistics show that the mortality amongst the unvaccinated patients was 40.91 per cent., whereas among the vaccinated it was 2.18 per cent. We have referred to the subject on a previous occasion; and we commend these official figures to the attention of those who do not believe in the efficacy of vaccination. The number of cases (seventy in all) is, of course, small, and of themselves the statistics are therefore of no great moment; but they are useful as adding one more link to the chain of evidence which demonstrates the usefulness of vaccination. The report further points out that at the Asylums Board hospitals revaccination of the members of the staff is compulsory, and that cases do not arise among them. But in Poor-Law infirmaries this rule is not enforced, and the result is seen in the epidemic at the Mile End infirmary: a single case admitted therein infected not only twenty-one of the other patients, but also seven of the staff.

### A Campaign against the House-fly.

The part played by the house-fly in spreading disease is one of those scientific observations which have caught the public fancy. It is perhaps not surprising to hear that a campaign against the domestic house fly has been in full swing in the United States, that it has been taken up with enthusiasm by householders, and that the small boy has proved his prowess in the probably congenial task of assisting in the extermination of the pest. Chicago appears to have aroused the American nation by starting and fomenting the campaign, and now we read of a great contest at Worcester, Massachusetts, in which over ten barrels of flies were gathered by the 232 contestants. It is estimated that the winner's contribution represented about 1,200,000 flies, and it is noteworthy that this champion fly-catcher is only twelve years old. Great improvement has taken place in many kitchens and restaurants, and throughout the country concerted measures are proving effective in reducing the numbers of these insects.

